# Ion mobility spectrometry as a simple and rapid method to measure the plasma propofol concentrations for intravenous anaesthesia monitoring

**DOI:** 10.1038/srep37525

**Published:** 2016-11-21

**Authors:** Xin Wang, Qinghua Zhou, Dandan Jiang, Yulei Gong, Enyou Li, Haiyang Li

**Affiliations:** 1 Key Laboratory of Separation Science for Analytical Chemistry, Dalian Institute of Chemical Physics, Chinese Academy of Sciences, Dalian, Liaoning, 116023, People’s Republic of China; 2 Department of Anesthesiology, The First Affiliated Hospital of Harbin Medical University, Harbin, Heilongjiang, 150001, People’s Republic of China

## Abstract

The plasma propofol concentration is important information for anaesthetists to monitor and adjust the anaesthesia depth for patients during a surgery operation. In this paper, a stand-alone ion mobility spectrometer (IMS) was constructed for the rapid measurement of the plasma propofol concentrations. Without any sample pre-treatment, the plasma samples were dropped on a piece of glass microfiber paper and then introduced into the IMS cell by the thermal desorption directly. Each individual measurement could be accomplished within 1 min. For the plasma propofol concentrations from 1 to 12 μg mL^−1^, the IMS response was linear with a correlation coefficient *R*^2^ of 0.998, while the limit of detection was evaluated to be 0.1 μg mL^−1^. These measurement results did meet the clinical application requirements. Furthermore, other clinically-often-used drugs, including remifentanil, flurbiprofen and atracurium, were found no significant interference with the qualitative and quantitative analysis of the plasma propofol. The plasma propofol concentrations measured by IMS were correlated well with those measured by the high performance liquid chromatography (HPLC). The results confirmed an excellent agreement between these two methods. Finally, this method was applied to monitor the plasma propofol concentrations for a patient undergoing surgery, demonstrating its capability of anaesthesia monitoring in real clinical environments.

Propofol, an intravenous anaesthetic agent, has been commonly used for the total intravenous anaesthesia in the surgery. In the clinical practice, the target controlled infusion (TCI) devices are increasingly used for the propofol administration. A plasma propofol concentration of 2 to 10 μg mL^−1^ is normally required for the induction of anaesthesia, while a concentration of 2 to 4 μg mL^−1^ should be administered continuously for the maintenance of the anaesthesia^1^. Whereas, the TCI models were developed with the data from healthy volunteers so that they might not be suitable for some special clinical situations^2^. In fact, sometimes the median absolute performance error of the TCI system was as high as 60%^3^. Therefore, measuring the plasma propofol concentrations during the anaesthesia is of great significance to enhance the safety of patients undergoing surgery.

So far, the high performance liquid chromatography (HPLC) is the most commonly used analytical method for measuring the propofol concentrations in plasma^4^,^5^,^6^,^7^,^8^,^9^,^10^,^11^, and it is also used as a reference to evaluate other methods^12^,^13^. However, due to the complex matrix of plasma, the complicated sample pretreatment must be implemented before the HPLC separation process can be carried out. Therefore, the required time duration for the plasma propofol measurement by HPLC is usually more than 30 min^1^,^5^,^6^. Such a delayed measurement result suggests that HPLC is not practical to provide the critically required measurement for the plasma propofol concentrations in a timely fashion during the clinical surgery operation, because the anesthetists do need such a propofol concentration information within a very short period of time, such as within 1 minute instead of more than a few dozen of minutes, in order to make a suitable adjustment for the propofol injection.

Ion mobility spectrometry (IMS) is a well-known technique for the separation and detection of gas phase ions in a weak electric filed, based on the differences in ion mobility at the atmospheric pressure. Featuring high sensitivity, fast analysis speed and suitable portability, IMS has been successfully used for the detection of explosives, illicit drugs, chemical warfare agents and toxic industrial compounds^14^,^15^,^16^,^17^,^18^,^19^,^20^,^21^. In recent years, IMS has also offered the great potential for breath analysis, such as the measurement of the propofol in exhaled air. In 2009, Perl *et al*.^22^ combined IMS with a multi-capillary column (MCC-IMS) to measure the exhaled propofol concentrations for the first time; afterwards, several related works were reported^23^,^24^,^25^, proving MCC-IMS a viable method for the exhaled propofol measurement. On the other hand, in our previous works, we constructed a membrane inlet for our own ion mobility spectrometry system using a hydrophobic silicone membrane, achieving a selective detection of the exhaled propofol^26^,^27^; subsequently, we developed a time-resolved dynamic dilution ion mobility spectrometry for measuring the exhaled air directly, realizing the anhysteretic monitoring of the exhaled propofol concentrations for the patients undergoing surgery^28^.

Therefore, it has been proved that IMS is an effective tool for the gaseous propofol measurement. However, no works have been published to use IMS measuring the propofol in liquid phase, especially for the plasma propofol. In this study, we demonstrated a stand-alone IMS to measure the propofol concentrations in plasma. The plasma samples were introduced into the IMS cell directly without any pre-treatment so that the analysis time was shortened significantly. The cross interference from other clinically-used drugs were investigated qualitatively and quantitatively. The proposed IMS method was evaluated by comparing its measurement results with those obtained by HPLC. Finally, this method was applied to monitor the plasma propofol concentrations for a patient undergoing surgery.

## Methods

Prior to the study, a protocol was approved by the Ethics Committee at Harbin Medical University (protocol no. 201314). The written informed consents were provided by all the participants who entered tests. All experiments were carried out in accordance with the approved guidelines.

The testing plasma was drawn from 10 healthy volunteers. Drugs including propofol, remifentanil, flurbiprofen and atracurium were provided by The First Affiliated Hospital of Harbin Medical University, China. Methanol used was of chromatographic grade and purchased from Kermel Chemicals Co., Ltd (Tianjin, China). A plasma stock with 100 μg mL^−1^ propofol was prepared by weighing and dissolving the propofol in the unspiked plasma, and then mixed with a liquid mixer for 1 min. The plasma samples with lower propofol concentrations were obtained by diluting the plasma stock with unspiked plasma.

**Figure 1:**
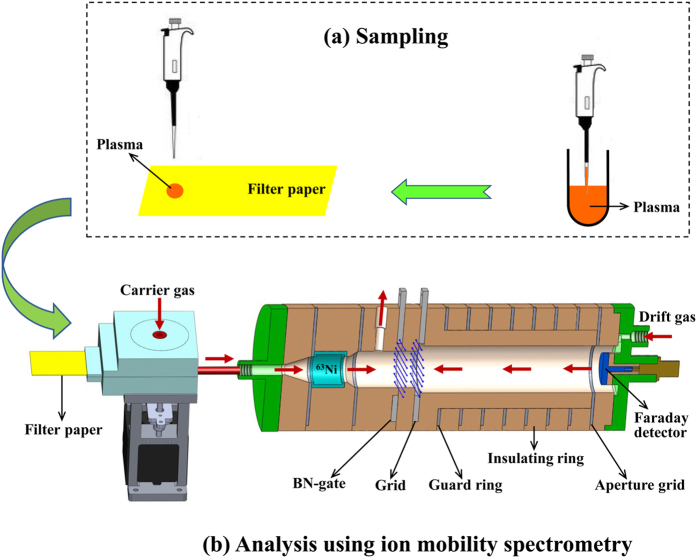
Schematic drawing of ion mobility spectrometry (IMS) for detecting propofol in plasma.

As shown in Fig. 1a, ^63^Ni-IMS apparatus with BN-grid structure was built for this research, while the design detail was the same as reported previously^29^. The IMS cell was running under an electric field of 374 V cm^−1^ at 100 °C. Clean air, filtered by the silica gel, activated carbon and 13X molecular sieve traps, was used as the carrier gas and drift gas for the IMS, with flow rates of 800 and 1000 mL min^−1^, respectively. The moisture of the purified air was kept below 1 ppm. In the tests, the plasma samples were introduced into the IMS cell by a thermal desorber, with detailed process steps as following: firstly, 20 μL plasma sample was deposited on a piece of glass microfiber paper (Grade GF/C, Whatman, UK); subsequently, the paper was inserted into the thermal desorber, where the vaporized sample molecules from the plasma were sent into the IMS cell by carrier gas.

The propofol concentration in plasma was also detected by a HPLC as a reference, where the propofol was detected by a UV detector working at 270 nm (JASCO1575). The HPLC mobile phase consisted 80% methanol and 20% water with a flow rate of 1.0 mL/min. For each 180 μL plasma sample, 20 μL of 530 μg mL^−1^ thymol solution (internal standard) and 800 μL methanol were added, and then the sample was mixed with a liquid mixer for 1 min. After the centrifugation process (14000 rpm for 15 min), 20 μL aliquots of the supernatant were injected into a 200 mm × 4.6 mm i.d. C_18_ silica gel column (Kromasil ODS, 5 μM) for separation and detection.

## Results and Discussions

### Identification of propofol in plasma

**Figure 2:**
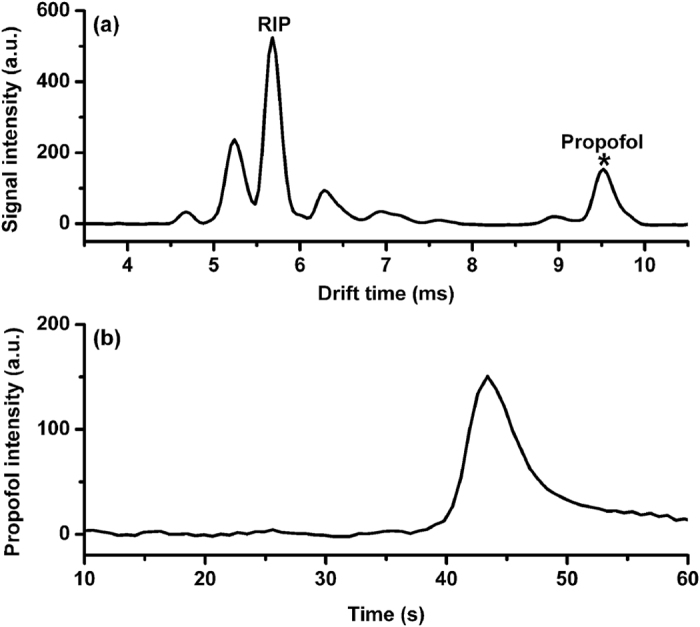
(**a**) Ion mobility spectrum of the plasma propofol, (**b**) the temporal profile of the propofol intensity in a single measurement.

Figure 2a displays the ion mobility spectrum for a concentration of 3.5 μg mL^−1^ propofol in the plasma, from which we observed a propofol ion peak with the drift time of 9.52 ms and the reduced mobility *K*_0_ of 1.39 cm^2^ V^−1^ s^−1^ (agrees with ref. 28). For each measurement, the peak intensity of the propofol was monitored as a function of time when the plasma sample was introduced, as depicted in Fig. 2b. The maximum intensity of this temporal profile, defined as *I*_max_, is dependent on the sensitivity of the IMS for the plasma propofol, while the time required to achieve the *I*_max_ is defined as *t*_max_ that can be used to characterize the analysis speed.

### Optimization of the thermal desorber temperature

**Figure 3:**
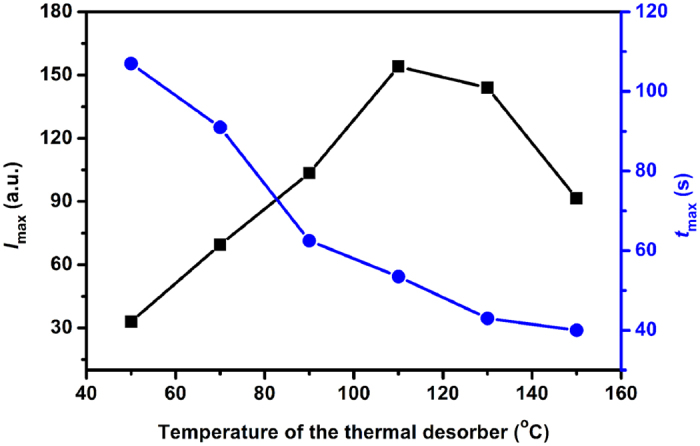
The effect of the thermal desorber temperature on *I*_max_ and *t*_max_.

With the thermal desorber temperature from 70 to 150 °C, we monitored a series of the temporal profiles for the plasma propofol, from which we obtained the effect of the thermal desorber temperature on *I*_max_ and *t*_max_, as illustrated in Fig. 3. It is clear that *I*_max_ increases initially and reaches the maximum at 110 °C, while the higher thermal desorber temperature brings a decreased *I*_max_. The initial increase of *I*_max_ should be attributed to the higher release efficiency of the propofol in the plasma; however, with a thermal desorber temperature higher than 110 °C, the other components in the plasma might be released at a significantly higher rate, which would consume the reactant ions in the IMS cell that lead to a decayed propofol signal. We observed that the higher thermal desorber temperature is, the higher release efficiency of the propofol in the plasma arises, and the shorter *t*_max_ becomes. Therefore, a thermal desorber temperature of 130 °C was selected for the following experiments. At this optimized temperature, an individual measurement can be accomplished within 1 min. This measurement process is much faster than that from other analyzers^12^,^13^.

### Linearity, limit of detection (LOD), and repeatability

**Figure 4:**
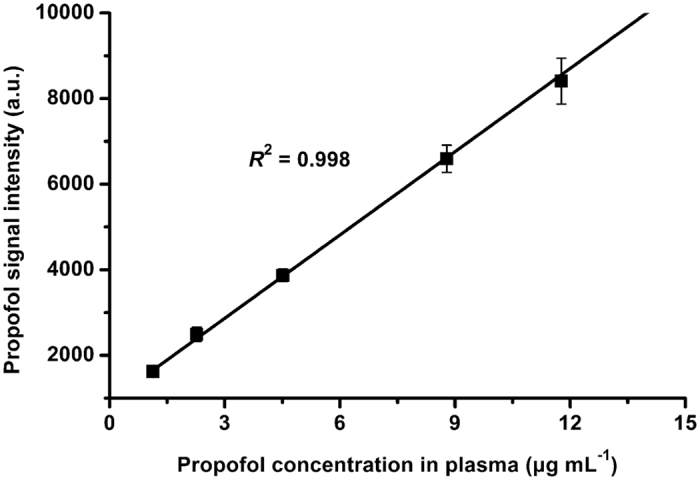
Linear response of ion mobility spectrometry (IMS) for the plasma propofol.

**Figure 5:**
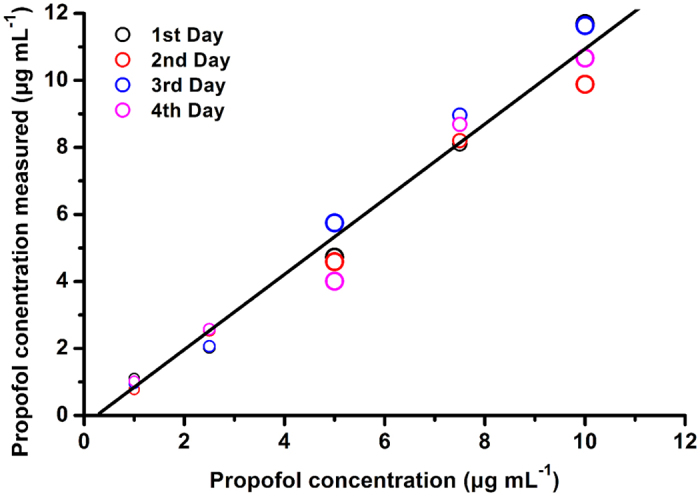
Various plasma propofol concentrations were measured by IMS for four times over one week period. The higher RSD of IMS measurement, the larger size of circles.

To investigate the linearity of the IMS for the plasma propofol, we measured five plasma samples spiked with clinically-used propofol concentrations from 1 to 12 μg mL^−1^. As depicted in Fig. 4, the result demonstrates an excellent linearity with a correlation coefficient *R*^2^ of 0.998. Based on the signal to noise ratio of 3, the LOD of IMS for the plasma propofol is calculated to be 0.1 μg mL^−1^. In Fig. 5, the plasma samples spiked with 1 to 10 μg mL^−1^ propofol were measured for four times over one week period, demonstrating an acceptable inter-day precision with the relative standard deviation (RSD) of 4.8 to 14.5%. This result could meet the clinical requirements for plasma propofol measurement during anaesthesia.

### Cross interference

**Figure 6:**
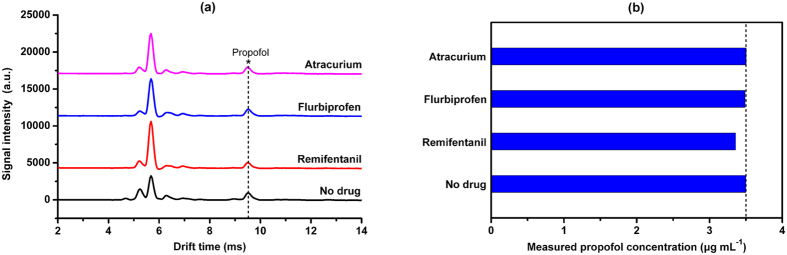
(**a**) Ion mobility spectra of the plasma propofol spiked with different interference drugs, (**b**) effect of interference drugs on the measured plasma propofol concentration.

In this test, the plasma samples, prepared to achieve the propofol concentration of 3.5 μg mL^−1^, were spiked with different clinically-used drugs, including 0.01 μg mL^−1^ remifentanil, 12.5 μg mL^−1^ flurbiprofen and 2.5 μg mL^−1^ atracurium, respectively. These plasma samples were measured by the IMS separately. Comparing with the unspiked control sample, no extra ion peaks in the ion mobility spectra was observed for these cross drugs, as shown in Fig. 6a. Furthermore, it is notable that the measured plasma propofol concentrations are basically independent from the cross drugs, as shown in Fig. 6b. Therefore, it can be concluded that the above cross drugs shows no significant interference with the qualitative and quantitative analysis of the propofol in the plasma.

### Method comparison

**Figure 7:**
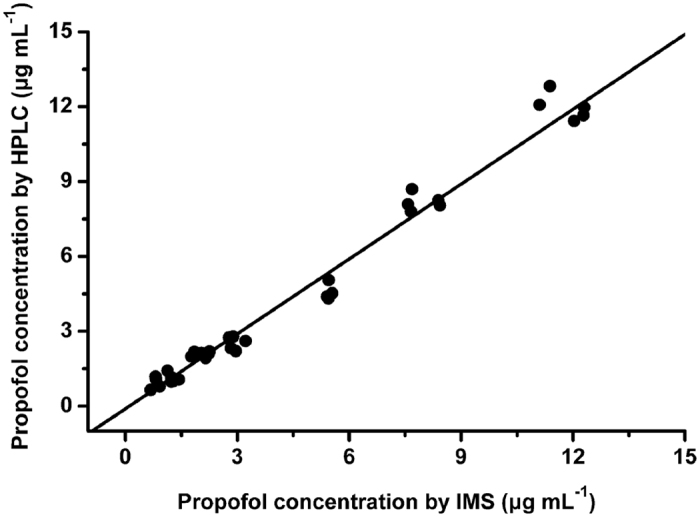
Correlation of the plasma propofol concentrations measured by IMS and the high performance liquid chromatography (HPLC).

**Figure 8:**
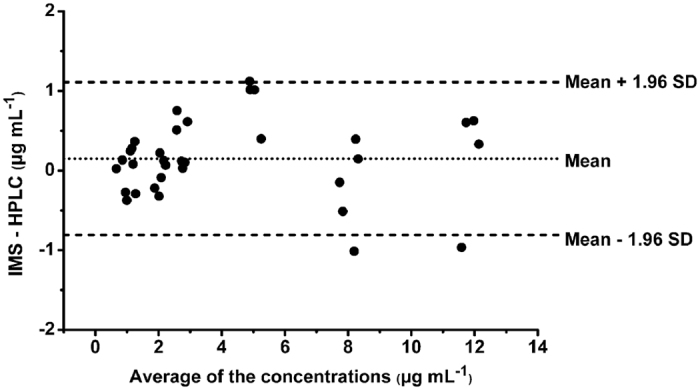
Blank-Altman plot showing the deviation of the plasma propofol concentrations measured by IMS and HPLC. Mean bias (0.15 μg mL^−1^) was shown with dotted line, mean ± 1.96 standard deviation (SD) limits were shown with dashed line.

A total of 34 plasma samples were prepared with the propofol concentrations from 1 to 10 μg mL^−1^, and then they were all measured by the IMS and HPLC, respectively. Figure 7 illustrates the scatter plot of the plasma propofol concentrations measured by these two methods, which suggests a linear relationship over the proposed concentration range. In Fig. 8, the agreement between these two methods is assessed using Bland-Altman method^12^. The result shows a small positive bias of 0.15 μg mL^−1^ (mean difference between the propofol concentration measured by the IMS and HPLC), with a standard deviation (SD) of 0.49 μg mL^−1^. According to these values, the 95% limits of agreement (mean ± 1.96 SD) are −0.81 to 1.11 μg mL^−1^, respectively.

### Clinical application

**Figure 9:**
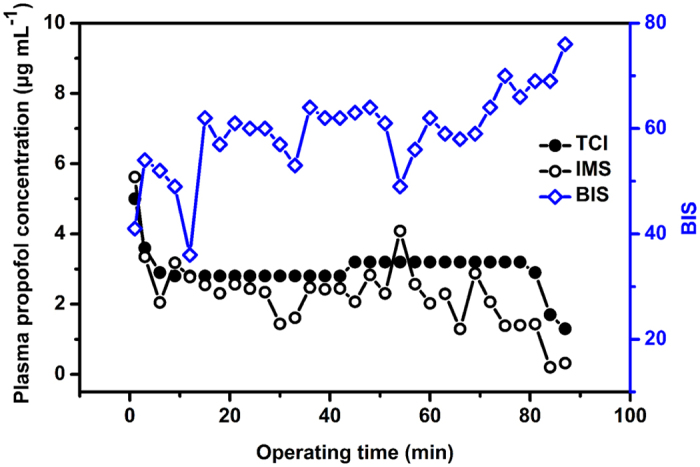
Temporal profiles of BIS values and plasma propofol concentrations measured by IMS and calculated by TCI system for a patient undergoing surgery.

Finally, we applied the proposed IMS method to monitor the plasma propofol concentrations for a patient undergoing surgery. As shown in Fig. 9, the plasma propofol concentration of TCI system was kept at 2.8 and 3.2 μg mL^−1^ during the anaesthesia maintenance, while the plasma propofol concentration measured by IMS was found to be oscillated. At present, the bispectral (BIS) index has been clinically used to monitor the anaesthesia depth. The lower BIS value indicates the deeper anaesthesia. In this clinical test, the BIS index was also monitored. Figure 9 shows the expected inverse development of BIS value and plasma propofol concentration measured by IMS, demonstrating the capability for anaesthesia monitoring in real clinical environments. Furthermore, we can find a hysteresis for the development of BIS value and plasma propofol concentration in Fig. 9. As each measurement of plasma propofol in this test was accomplished with an interval of 3 min, which made it difficult to identify the hysteresis time accurately. Thus, the frequency of sampling plasma should be increased in future works, so that the relation of plasma propofol concentration with BIS value can be investigated accurately.

## Conclusions

In this work, we constructed a stand-alone ^63^Ni-IMS apparatus to measure the plasma propofol concentrations without any sample pre-treatment. The measurement reliablity of the IMS results was demonstrated using HPLC as the reference method. Our results illustrated that both the LOD and linearity of the IMS for the propofol met the clinical measurement requirements. More significantly, an individual IMS measurement can be accomplished within 1 min so that this process is rapid enough to provide the anaesthetists with the plasma propofol concentrations in real time. Therefore, this work promises a simple and rapid method for the intravenous anaesthesia monitoring in real time to help the anaesthetists achieve an accurate anaesthesia levels for the patients undergoing surgery in the clinical environment.

## Additional Information

**How to cite this article**: Wang, X. *et al*. Ion mobility spectrometry as a simple and rapid method to measure the plasma propofol concentrations for intravenous anaesthesia monitoring. *Sci. Rep.* **6**, 37525; doi: 10.1038/srep37525 (2016).

**Publisher’s note:** Springer Nature remains neutral with regard to jurisdictional claims in published maps and institutional affiliations.
